# Strengthening Nutrition Interventions in Antenatal Care Services Affects Dietary Intake, Micronutrient Intake, Gestational Weight Gain, and Breastfeeding in Uttar Pradesh, India: Results of a Cluster-Randomized Program Evaluation

**DOI:** 10.1093/jn/nxab131

**Published:** 2021-05-26

**Authors:** Phuong H Nguyen, Shivani Kachwaha, Lan M Tran, Rasmi Avula, Melissa F Young, Sebanti Ghosh, Praveen K Sharma, Jessica Escobar-Alegria, Thomas Forissier, Sumeet Patil, Edward A Frongillo, Purnima Menon

**Affiliations:** International Food Policy Research Institute, Washington, DC, USA; International Food Policy Research Institute, Washington, DC, USA; FHI Solutions, Washington, DC, USA; International Food Policy Research Institute, Washington, DC, USA; Hubert Department of Global Health, Emory University, Atlanta, GA, USA; FHI Solutions, Washington, DC, USA; FHI Solutions, Washington, DC, USA; FHI Solutions, Washington, DC, USA; FHI Solutions, Washington, DC, USA; Network for Engineering, Economics, Research and Management (NEERMAN), Delhi, India; Health Department of Promotion, Education, and Behavior, Arnold School of Public Health, University of South Carolina, Columbia, SC, USA; International Food Policy Research Institute, Washington, DC, USA

**Keywords:** maternal nutrition, diet quality, micronutrient intake, breastfeeding, interpersonal counseling, India

## Abstract

**Background:**

Maternal nutrition interventions are inadequately integrated into antenatal care (ANC). Alive & Thrive aimed to strengthen delivery of micronutrient supplements and intensify interpersonal counseling and community mobilization through government ANC services.

**Objectives:**

We compared nutrition-intensified ANC (I-ANC) with standard ANC (S-ANC) on coverage of nutrition interventions and maternal nutrition practices.

**Methods:**

We used a cluster-randomized design with cross-sectional baseline (2017) and endline (2019) surveys (*n* ∼660 pregnant and 1800 recently delivered women per survey) and a repeated-measures longitudinal study in 2018–2019 (*n* = 400). We derived difference-in-difference effect estimates (DIDs) for diet diversity, consumption of micronutrient supplements, weight monitoring, and early breastfeeding practices.

**Results:**

Despite substantial secular improvements in service coverage from India's national nutrition program, women in the I-ANC arm received more home visits [DID: 7–14 percentage points (pp)] and counseling on core nutrition messages (DID: 10–23 pp) than in the S-ANC arm. One-third of women got ≥3 home visits and one-fourth received ≥4 ANC check-ups in the I-ANC arm. Improvements were greater in the I-ANC arm than in the S-ANC arm for any receipt and consumption of iron–folic acid (DID: 7.5 pp and 9.5 pp, respectively) and calcium supplements (DID: 14.1 pp and 11.5 pp, respectively). Exclusive breastfeeding improved (DID: 7.5 pp) but early initiation of breastfeeding did not. Maternal food group consumption (∼4 food groups) and probability of adequacy of micronutrients (∼20%) remained low in both arms. Repeated-measures longitudinal analyses showed similar results, with additional impact on consumption of vitamin A–rich foods (10 pp, 11 g/d), other vegetables and fruits (22–29 g/d), and gestational weight gain (0.4 kg).

**Conclusions:**

Intensifying nutrition in government ANC services improved maternal nutrition practices even with strong secular trends in service coverage. Dietary diversity, supplement consumption, and breastfeeding practices remained suboptimal. Achieving greater behavior changes will require strengthening the delivery and use of maternal nutrition services integrated into ANC services in the health system. This trial was registered at clinicaltrials.gov as NCT03378141.

## Introduction

Maternal undernutrition is an important public health concern both globally and in India. Consequences associated with maternal undernutrition include the mortality and morbidity burden for mothers and their children, specifically, fetal growth restriction, stunting, wasting, nutrient deficiencies, and neonatal deaths. About 3.1 million global child deaths (1) and 68% of under-5 child deaths in India (2) are attributable to maternal and child undernutrition. There is potential to reduce such deaths by 15% if certain nutrition-focused interventions reach populations at 90% coverage (3). If evidence-based interventions are provided effectively to those already seeking care, an estimated 28% of maternal and neonatal deaths and 22% of stillbirths could be prevented (4).

Despite global understanding on the importance of reducing maternal undernutrition, it remains highly prevalent, particularly in South Asia and India (5), which carries one-third of the global burden of undernutrition. The prevalence of maternal low BMI (BMI <18.5 kg/m^2^) is 10%–20% globally but 30%–40% in South Asia and India (5, 6). About one-third of pregnant women globally are anemic (7), with the highest incidence of anemia cases in South Asia (8). In India, more than half of pregnant and nonpregnant women are anemic, and progress to reduce anemia has been slow in the last decade (9).

Maternal nutrition interventions can be delivered through several platforms; among them, health systems are considered the most effective platform to reach women and children in the first 1000 d (10). Gaps in coverage and inconsistent quality of counseling on maternal nutrition, however, have hindered the improvement of behavioral outcomes associated with the services (11). In 2016, the WHO issued new guidelines on antenatal care (ANC) for a positive pregnancy experience (12), but evidence-based interventions to improve maternal nutrition are currently poorly integrated in ANC services globally (3). The coverage of most nutrition interventions falls far below the coverage of the health services through which they are delivered, particularly for ANC, where the coverage of iron–folic acid (IFA) supplement consumption was about half of ANC (10). Similar missed opportunities were also found for India, where in 2016 the coverage of ANC was 51%, but IFA supplement consumption was lower (39% consumed ≥90 tablets and 31% ≥100 tablets) (13). These missed opportunities were even more pronounced in Uttar Pradesh, the second-largest state in India with a population >200 million and an annual birth cohort of >5 million, where only 26% of the women received ≥4 ANC visits and only 13% consumed IFA tablets for ≥100 d (14). This low coverage suggests that there is a need to fully utilize the existing contacts to deliver maternal nutrition interventions within maternal, newborn, and child health services. Closing the opportunity gap by increasing nutrition intervention coverage among those already reached by health services should be an immediate priority. Evidence is lacking on how maternal nutrition interventions can integrate effectively into existing government ANC services.

Alive & Thrive (A&T) is an initiative that supports scaling up of nutrition interventions to save lives, prevent illnesses, and contribute to healthy growth and development through improved maternal nutrition and infant and young child feeding practices in several countries. In India, A&T aimed to address the challenges of maternal undernutrition through integrating nutrition-focused social and behavior change communication (SBCC) and systems-strengthening interventions into existing government Reproductive, Maternal, Neonatal, Child, and Adolescent Health (RMNCH+A) services to intensify the nutrition content of ANC contacts. We hypothesized that the nutrition-intensified ANC (I-ANC) would have greater coverage of nutrition interventions, consumption of IFA and calcium supplements, diet diversity, and early breastfeeding practices than the standard ANC (S-ANC). We evaluated these hypotheses using a cluster-randomized evaluation that included both repeated cross-sectional surveys to assess population-level impact and individual repeated-measures surveys.

## Methods

### Study context and intervention description

In India, standard RMNCH+A programs are delivered through 2 major national programs: *1*) the Integrated Child Development Services (ICDS) managed by the Ministry of Women and Child Development; and *2*) the National Health Mission managed by the Ministry of Health and Family Welfare. Whereas food supplementation and growth monitoring interventions are delivered through the ICDS program, micronutrient supplementation, deworming, health check-ups, and curative interventions are delivered through the National Health Mission, and behavior change communication is delivered through both the programs (15, 16). Routine services are delivered by 3 types of frontline workers (FLWs)—Anganwadi workers (AWWs), Accredited Social Health Activists (ASHAs), and Auxiliary Nurse Midwives (ANMs)—through different platforms including home visits, ANC check-ups, Village Health and Nutrition Days (VHNDs), and community-based events.

This study (NCT03378141) was conducted in the context of POSHAN Abhiyaan, India's flagship National Nutrition Mission, which was rolled out in both I-ANC and S-ANC areas during the implementation period (2018–2019), aiming to improve maternal and child nutritional outcomes and ensure service delivery through capacity building, leveraging technology, behavioral change communication and community mobilization, and cross-sectoral convergence (17). Under POSHAN Abhiyaan, capacity-building activities for FLWs occur through the incremental learning approach (ILA) (18). ILA uses learning-by-doing to build the capacity and motivation of FLWs through monthly training on 1 specific topic, followed by 1 mo of practice to follow up on actions related to the topic. The ILA content includes 21 modules focused on maternal and child health and nutrition issues. POSHAN Abhiyaan also includes strategies of behavior change communication and community mobilization. Community-based events are held nationwide in March and September to carry out media campaigns for awareness generation on 12 key themes related to ANC, breastfeeding, anemia, complementary feeding, and water and sanitation. Multiple stakeholders are involved to ensure widespread participation such as the ministries of health and sanitation, schools, self-help groups, and village organizations. District authorities were made responsible for program implementation and periodic reviews. The Anemia-Free India campaign also rolled out in this period focused on anemia prevention and management, including activities on improving the supply chain of IFA supplements, anemia testing, training of FLWs, and SBCC initiatives.

A&T aimed to facilitate prioritization and strengthening of maternal nutrition services through the existing government ANC platform by means of 6 activities: *1*) capacity building for FLWs and supervisors; *2*) providing technical support to intensified maternal nutrition service provision including interpersonal counseling and improving quality contacts during pregnancy; *3*) expanded community mobilization to improve awareness and shift norms and perceptions; *4*) strengthening supportive supervision; *5*) strengthening of the IFA and calcium supplementation supply chain; and *6*) strategic use of data to track the progress of interventions and identify areas needed for improvement. The A&T maternal nutrition interventions were implemented in I-ANC areas from June 2018 to December 2019, reaching ∼16,000 women over 18 mo (19) and operating in the same time frame as POSHAN Abhiyaan to strengthen systems for nutrition intervention delivery. The key additional inputs by A&T in I-ANC areas beyond POSHAN Abhiyaan activities were as follows: additional trainings and materials on maternal nutrition, supply chain management for supplement distribution, strengthening use of data to monitor program progress, encouraging supportive supervision practices, and additional community mobilization through husbands’ forums. **Table 1** gives details of the content, frequency, quality, and differences between I-ANC and S-ANC areas.

### Evaluation design

The impact evaluation design used a pair-matched clustered randomized design, with repeated cross-sectional surveys at baseline (2017) and endline (2019). Thirteen pairs were formed of 26 blocks from 2 districts (Kanpur-Dehat and Unnao) in Uttar Pradesh based on several demographic, infrastructure, and amenity characteristics taken from Census 2011 data. One block from each pair was randomly assigned either to I-ANC (13 blocks) or S-ANC (13 blocks). Cross-sectional household surveys were conducted in the same set of 182 randomly sampled Gram Panchayats (typically a group of 1–3 villages but henceforth referred to as villages) at the same time of year (November–December, harvest season in the survey areas) for both baseline and endline; therefore, the design is longitudinal at the village level and focused on the public health and implementation systems impact.

To complement the repeated cross-sectional design and provide additional insights on individual-level impacts, we also implemented a repeated-measures longitudinal study which followed the same 2-arm cluster-randomized design, with repeated surveys of newly recruited pregnant women (*n* = 496 women, 251 in I-ANC and 245 in S-ANC) from the first trimester until 42 d postpartum. These women were selected from 76 randomly sampled villages that were in the same 26 blocks but separate from the 182 villages included in the repeated cross-sectional design. The samples for the 2 designs were intentionally kept distinct to ensure that impact evaluation findings were not influenced by the repeated measurements planned in this design.

### Sample size estimations

There were 2 samples of interest for the study outcomes: *1*) recently delivered women with children <6 mo of age, which provided the best opportunity to assess most of the outcome indicators; and *2*) pregnant women in the second and third trimesters of pregnancy, which allowed for assessment of dietary diversity during pregnancy. Sample sizes were estimated based on the expected prevalence of the primary outcomes (i.e., IFA supplement consumption and dietary diversity), expected change after the intervention, power to detect those differences at 0.80, testing at an α of 0.05, and intraclass correlations of 0.03–0.05 for different outcomes based on previous data in India (6). Assuming the percentage of women consuming ≥100 IFA tablets during pregnancy was 13% at baseline based on National Family Health Survey (NFHS)-4 data for Uttar Pradesh (6), we estimated that a minimum of 900 women/group would be needed to detect a difference of 12 percentage points (pp) between intervention areas in the changes over time. This sample would also allow detecting a group difference of 10 pp for changes in early initiation of breastfeeding from a baseline of 25.2% (6). In addition, 300 pregnant women/group would be sufficient to detect a 10-pp difference in women consuming ≥5 food groups/d [assuming the prevalence of women who had minimum dietary diversity at baseline was 9% (6)]. These detectable differences are of public health importance.

For the repeated-measures longitudinal design, we estimated that a minimum of 200 women/group would have ≥80% power to detect the aforementioned difference between intervention areas in the changes over time. Allowing a 10% buffer in the sample, a total sample of 440 pregnant women (220/group) would be needed.

### Randomization

A situational analysis of all blocks in the 2 districts was conducted, assessing several factors associated with maternal and child health and nutrition based on Census 2011 data (20) and NFHS-4 data (6). These factors included socioeconomic profile; availability of health and nutrition centers; use of health, nutrition, and agricultural services; education level; access to water and sanitation; participation in social welfare programs; nutrition outcomes; and determinants. A propensity score matching method was used to identify pairs of intervention and control blocks (21), and then we randomly assigned them to either the intervention (13 blocks) or the control (13 blocks) group through a manual lottery. The randomization process was carried out in the presence of representatives from government offices, A&T, and IFPRI staff. There was no blinding of the interventions at the level of service delivery.

**TABLE 1:** Description of differences between I-ANC and S-ANC intervention areas in Uttar Pradesh, India^1^

Interventions	I-ANC areas	S-ANC areas
Capacity building		
Training of FLWs and supervisors	- As part of the National Nutrition Mission program, FLWs in both areas received training on maternal and child nutrition through the incremental learning approach mechanism	
	- Convergent actions were encouraged and performance incentives were included	
	- FLWs receive a full-day technical training on MN content (diet diversity, IFA and calcium supplementation, weight gain, and breastfeeding) and counseling approach with pregnant women with follow-up training/orientation during monthly meetings	- Routine training and supervision activities without additional performance support
	- Supervisors receive 2 full-day trainings on MN content and on supporting FLWs to deliver MN services as well as use of data	
	- FLWs received hands-on mentoring during home visits to help beneficiaries translate the skills learnt into practice	
	- FLWs and supervisors receive SBCC materials on MN and job aid to retain training content	- Standard materials
MN services		
Overall MN services	- Under the national mission, in both I-ANC and S-ANC areas, existing programs on safe pregnancy and access to ANC (22) and promotion of breastfeeding (23) were rejuvenated. In addition, the Anemia-Free India program (24), also launched as part of the nutrition mission, renewed focus on the distribution of prophylactic IFA and deworming along with increased diagnostics and curative approaches for treating anemia	
Counseling on dietary diversity and quantity	- Intensified interpersonal counseling/coaching/demonstrations during home visits for pregnant women and family members using local foods by ASHAs and AWWs	- Routine standard counseling
	- Reminder messages at VHNDs/ANC contacts at subcenters by ANMs and by facility-based service providers	
	- Peer group problem-solving discussions at AWC group meetings	
IFA and calciumsupplementationand counseling	- Intensified distribution of 180 IFA and 360 calcium tablets during pregnancy	- Routine IFA and calcium distribution to pregnant women
	- Intensified interpersonal counseling at home visits for pregnant women and family members with emphasis on benefits, dosage, and adherence/compliance, by ASHAs and AWWs	- Routine standard counseling
	- Counseling/reminder messages on dosage and adherence/compliance, by ANMs at VHNDs and subcenters and by facility-based service providers	
Weight measurementin pregnancy	- Functional weighing scale at AWC and VHNDs	- Standard materials
	- Intensified interpersonal counseling during home visit on importance of adequate weight gain by ASHAs and AWWs	- Routine standard counseling
	- Weight gain chart at home for women and family members	
	- Weight monitoring and reminder messages at VHNDs and subcenters by ANMs and other facility-based providers	
Counseling on breastfeeding	- Focus on counseling by ANMs during VHNDs and at subcenters and by other facility-based providers during ANC (second- and third-trimester visits) on early initiation of breastfeeding	- Routine counseling on early breastfeeding
Number of ANC and home visits during pregnancy	- Optimizing the existing mandated contact by improving quality through intensified counseling (minimum 30 min per counseling session) and demonstration	- Routine training/performance improvement support for quality home visits/contacts by FLWs
	- 4 minimum quality contacts with ANMs for ANC services at VHNDs or subcenters (1 each during the first and second trimesters and 2 during the third trimester)	
	- 4 minimum quality contacts by ASHAs (1 each during the first and second trimesters and 2 during the third trimester) and 3 minimum contacts by AWWs (1 during the second trimester and 2 during the third trimester) for home visits to ensure pregnant women seek antenatal care at VHNDs and coaching/counseling/checking compliance/recommended practices	
Community mobilization		
Community events and outreach	- In both I-ANC and S-ANC areas, community awareness-raising activities were conducted periodically because community mobilization was a key pillar of the National Nutrition Mission	
	- Community mobilization involved key influencers in families (husbands, mothers/mothers-in-law), local community leaders, and Village Health, Sanitation, and Nutrition Committee members, aiming to improve awareness and shift norms and perceptions related to MN	- Routine community activities
	- Husbands’ forums (1/village during the intervention period)	
	- Community sensitization sessions with Village Health, Sanitation, and Nutrition Committee members and local community leaders (monthly meeting)	
	- Sensitization of local doctors in the public health system	
Supportive supervision		
Observation of MN counseling and services	- Government supervisors and A&T staff conduct supervision visits to FLWs at different service-delivery contact points (home visits, VHNDs, community-based events, etc.)	- Standard supervision without additional performance improvement support
	- Supervisors were encouraged to adopt a supportive supervision checklist, provide coaching, use a problem-solving method, and provide feedback to FLWs for performance improvement as part of their regular supervision visits	
	- Supportive supervision activities were reviewed during monthly meetings based on information from the checklists	
Supply chain strengthening		
Supplies of IFA and calcium supplements	- Under the national Anemia-Free India program, activities to strengthen the IFA supply chain were undertaken in both I-ANC and S-ANC areas	
	- Distribution of 180 IFA and 360 calcium tablets was intensified in I-ANC areas	- Routine activities related to the supply chain
	- A&T staff provided technical support to facilitate accurate forecasting and procurement of adequate quantities of IFA and calcium at state and district levels	
	- Block staff and ANMs also received technical support to improve demand estimation and rational distribution based on requirements for IFA and calcium supplements	
Use of data		
Strategic use of data for program improvement	- A&T staff supports ANMs to check data quality and make necessary revisions during reporting for the Mother and Child Tracking system	- Routine monitoring of activities or use of data
	- A&T staff collects program monitoring data on MN activities including home visits, VHNDs, husbands’ forums, and review meetings to monitor coverage and quality	
	- A&T supports government staff to develop and use MN block cards using the Health Management Information System to monitor health and nutrition indicators each month	
	- Data discussed in review meetings to identify gaps and areas for improvement	

^1^ANC, antenatal care; ANM, Auxiliary Nurse Midwife; ASHA, Accredited Social Health Activist; AWC: Anganwadi Center; AWW, Anganwadi worker; A&T, Alive & Thrive; FLW, frontline worker; I-ANC, nutrition-intensified antenatal care; IFA, iron and folic acid; MN, maternal nutrition; S-ANC, standard antenatal care; SBCC, social and behavior change communication; VHND, Village Health and Nutrition Day.

From each block, 7 villages were selected using probability proportional to size, yielding a total of 182 villages. A household listing was conducted for each village to create a sample frame of pregnant women and mothers with infants <6 mo of age. Using systematic random sampling, we selected 4 households with pregnant women and 13 households with children 0–6 mo old. If the village had less than the required sample, we compensated for that by linking the nearest neighboring village. Women who could not understand and answer questions were excluded as part of the informed consent process. Randomization was conducted for the cross-sectional survey first from the overall sampling frame and then for the repeated-measures survey from the remaining clusters following the same procedures.

### Ethical approval

This study was approved by the ethical review board from the International Food Policy Research Institute and the Suraksha Independent Ethics Committee in India. Verbal informed consent was obtained from all participants. The overall Alive & Thrive systems-strengthening efforts to improve maternal nutrition programming were approved by the Ministry of Health and Family Welfare, Government of India (DO No. M.12015/55/2016-MCH), and additional permissions for the evaluation data collection were provided by the State Government of Uttar Pradesh.

### Data collection

Data were collected via face-to-face interviews using a structured questionnaire which was prepared in English and translated into Hindi. Enumerators were recruited locally and were trained on technical content as well as security and confidentiality issues by mixed methods (lecture, role-play, mock interview, and practice) in a classroom and field settings. Field supervisors received additional training related to quality control processes; cross-checking, editing, and coding of the questions; and security and confidentiality issues.

Coverage and use of maternal nutrition interventions were assessed by asking recently delivered women about ANC services (whether they received ANC visits, the timing of the first visit, and the total number of visits), home visits (whether they were visited at home by different types of FLWs and how many times), VHNDs (whether they participated in VHNDs and how many times during the last 3 mo), and counseling received on maternal nutrition during pregnancy and breastfeeding.

Receipt and consumption of IFA and calcium supplements were assessed among recently delivered women by asking whether they received IFA and calcium supplements for free or purchased them, and the amount they received and consumed during their last pregnancy. Each woman in I-ANC areas was also provided a maternal nutrition calendar where AWWs and ASHAs could help record the number of IFA and calcium tablets consumed during monthly home visits; this calendar was used to assist women in their recall. Empty strips or bottles of IFA and calcium tablets were also used by enumerators to assist in the recall.

Dietary intake was assessed among pregnant women using a multiple-pass quantitative 24-h recall. Women were asked to describe all the foods and beverages they consumed during the previous day and night (both inside and outside the home), as well as the time of consumption, cooking method, and portion size. All reported foods were recategorized into the 10 food groups based on the Minimum Dietary Diversity for Women measurement guide (25) and dietary diversity was defined as consuming ≥5 food groups/d. To estimate the distribution of usual intake and account for within-person variation, a subset of pregnant women (10%) was selected to have two 24-h recalls ≥2 d apart.

Nutrient intakes were calculated using Indian food composition tables (26). Estimated usual intakes for each woman were used to calculate probabilities of adequacy for individual micronutrients (27, 28). The mean probability of adequacy (MPA) of each woman's intake was computed as the average of the probabilities of adequacy for a set of 11 micronutrients: calcium, iron, zinc, vitamin C, thiamin, riboflavin, niacin, vitamin B-6, folate, vitamin B-12, and vitamin A (29).

Early breastfeeding practices were assessed among recently delivered women with infants <6 mo old based on the WHO-recommended indicators (30), including *1*) early initiation of breastfeeding (i.e., proportion of infants put to the breast within 1 h of birth) and *2*) exclusive breastfeeding in the previous 24 h (i.e., proportion of infants <6 mo old who consumed only breast milk).

Covariates possibly associated with uptake of interventions or behavioral outcomes were also measured. Child variables were child age and sex. Maternal variables measured were mother's age, religion, education, occupation, and caste categories. Household variables measured were household size, number of children <5 y old, food security [measured using the Household Food Insecurity Access Scale (31)], and socioeconomic status (created by principal components analysis using variables on ownership of house and land, housing quality, access to services, and household assets) (32).

Recognizing the potential role of social desirability in influencing reporting of nutrition behaviors, we administered 12 questions to assess social desirability bias (33). These questions assessed the tendency of respondents to answer questions in a manner that is viewed favorably by others (**Supplemental Table 1**). Social desirability data were used to assess if results for primary outcomes differed by social desirability status.

For the repeated-measures study, data were collected in 3 main rounds: early pregnancy (<4 mo), late pregnancy (≥7 mo), and within 42 d of delivery. Data collection included information on exposure to ANC and postnatal care as well as outcomes on maternal nutrition practice. In addition, women were tracked by 3 or 4 short surveys separated by ∼1 mo (between early and late pregnancy) to track key nutritional practices and exposure to the interventions. This tracking of pregnant women allowed complementing self-reported data with data from observations of food availability in the household, counting of IFA and calcium strips, and monthly weighing during pregnancy.

### Statistical analysis

Data were analyzed on an intention-to-treat basis. The baseline characteristics of the study participants were compared between the 2 study areas, using linear (continuous variables) or logit (categorical variables) regression models. For repeated cross-sectional surveys, impacts were estimated using regression models that estimated differences in changes over time between the 2 study areas (34). The analysis was implemented using the Stata *diff* command accounting for pairing before randomization and clustering at the village level with a cluster version of the Huber–White (i.e., sandwich) robust estimator of SEs and using df appropriate for the number of blocks given that the arms were allocated to blocks (35). The difference-in differences were estimated based on 3 assumptions: *1*) allocation of intervention was not determined by outcomes, *2*) the difference between the I-ANC and S-ANC arms was constant over time in the absence of interventions, *3*) the composition of the intervention and comparison arms was stable for a repeated cross-sectional design. The fixed effects in the 2-level regression model were arm, survey, and arm × survey; the latter estimates the difference between arms in changes over surveys. Models for pregnant women's dietary intake adjusted for gestational age; models for breastfeeding practices adjusted for infant age and sex.

Data from different rounds of the repeated-measures design were used to complement the main impact estimates. Data were analyzed on an intention-to-treat basis using the *mixed* procedure. For continuous outcomes (such as number of food groups or number of IFA tablets consumed), 3-level (i.e., cluster, individual, residual) mixed-effects linear models were used, accounting for clustering at the block level and at the individual level as random effects (36). For dichotomous variables (such as percentage that consumed ≥100 IFA tablets or percentage that consumed ≥5 food groups), a linear model was used after first affirming that the percentages were not close to 0% or 100% given that 3-level mixed logistic models may not be able to be estimated. The interaction of arm (I-ANC compared with S-ANC) and round was used to test for differences between arms in the changes from recruitment to the final follow-up round.

All analyses were performed using Stata version 16.0 (StataCorp LP). A statistical analysis plan was developed before endline data collection, agreed upon among researchers of the evaluation team, and shared with the implementation team.

## Results

### Trial profile

**FIGURE 1:**
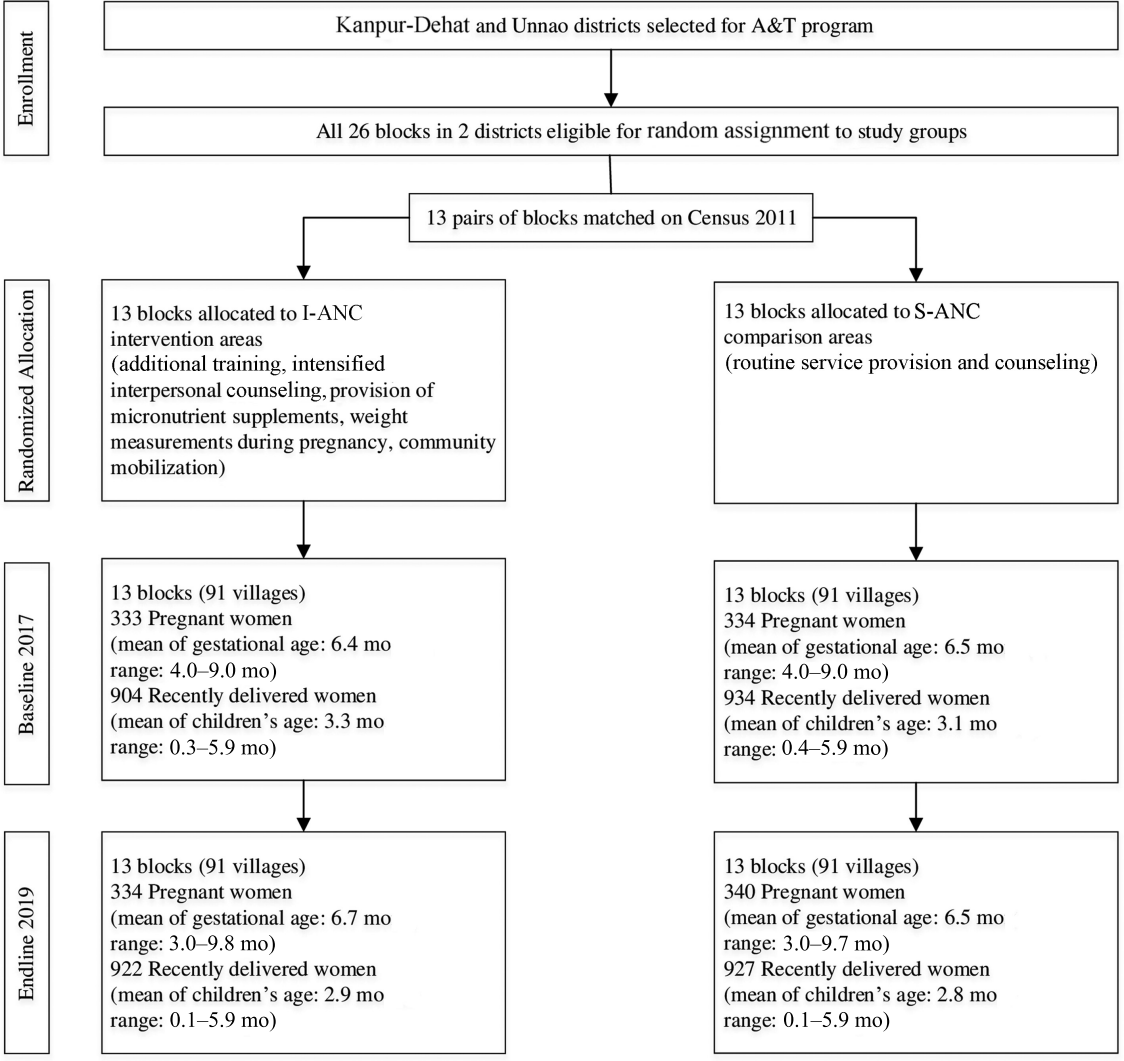
Trial profile from repeated cross-sectional surveys. A&T, Alive & Thrive; I-ANC, nutrition-intensified antenatal care; S-ANC, standard antenatal care.

No evaluation clusters were lost to follow-up, and none crossed from S-ANC to I-ANC areas during implementation (**Figure 1**). The cluster size was similar across clusters and over time. The individual repeated-measures longitudinal study experienced some loss to follow-up due to miscarriages, mother or child deaths, refusals, migrations, or early deliveries; this was similar between the 2 areas (**Supplemental Figure 1**).

### Sample characteristics

There were no differences in cluster, maternal, and household characteristics between the intervention and comparison areas at baseline (**Table 2**). On average, women were 25–26 y old and ∼90% were housewives. About one-third of the women had completed high school or above, whereas 24%–30% had no formal schooling. Food insecurity was experienced by 17% of households with pregnant women and 27% of households with recently delivered women. Social desirability bias scores were similar for both arms at endline (Supplemental Table 1).

**TABLE 2:** Selected baseline characteristics of pregnant and recently delivered women in I-ANC and S-ANC intervention areas in Uttar Pradesh, India^1^

	Pregnant women		Recently delivered women	
	I-ANC area (*n* = 333)	S-ANC area (*n* = 334)	I-ANC area (*n* = 904)	S-ANC area (*n* = 934)
Maternal characteristics				
Gestational age, mo	6.4 ± 1.4	6.5 ± 1.4	—	—
Second trimester, %	39.0	33.8	—	—
Third trimester, %	61.0	66.2	—	—
Age of respondent mother, y	25.2 ± 4.1	24.9 ± 4.1	25.8 ± 4.2	25.8 ± 4.4
Education level, %				
Never attended school, %	23.7	27.8	28.2	31.1
Primary school (grade 1–5), %	15.9	14.7	13.9	14.8
Middle school (grade 6–9), %	21.9	19.2	22.6	21.3
High school (grade 10–12), %	27.0	22.8	23.5	24.8
Graduate and above, %	11.4	15.6	11.8	7.9
Occupation as housewife, %	91.9	91.6	89.4	87.8
Religion as Hindu, %	92.2	91.9	94.2	92.3
Caste category, %				
Scheduled caste/tribe, %	41.1	42.2	39.3	42.7
Other backward class, %	43.5	39.5	44.4	43.9
General/others, %	15.3	18.3	16.4	13.4
BMI, kg/m^2^	—	—	21.0 ± 3.1	20.8 ± 2.9
Underweight (<18.5), %	—	—	21.5	20.3
Normal (18.5–24.9), %	—	—	68.4	72.4
Overweight/obese (≥25), %	—	—	10.2	7.3
Household characteristics				
Household size, *n*	5.1 ± 2.1	5.2 ± 2.0	6.4 ± 2.4	6.4 ± 2.4
Children <5 y, *n*	0.8 ± 0.7	0.7 ± 0.7	1.7 ± 0.7	1.7 ± 0.7
Household food secure, %	83.1	83.5	74.7	71.2
Household socioeconomic index	−0.02 ± 0.9	0.02 ± 0.9	0.05 ± 1.0	−0.05 ± 0.9
Tertile 1, %	33.3	33.5	31.4	35.2
Tertile 2, %	35.4	31.1	33.5	33.2
Tertile 3, %	31.2	35.3	35.1	31.6

1 Values are means ± SDs or percentages. I-ANC, nutrition-intensified antenatal care; S-ANC, standard antenatal care.

### Effects on coverage and use of ANC and nutrition services

At endline, most women (∼90%) received ANC during pregnancy (**Table 3**), but less than half of women (41%–44%) received ANC in the first trimester of pregnancy and only one-fourth received ≥4 ANC check-ups, with no difference between the 2 study arms. Home visits by AWWs and ASHAs improved in both areas from baseline with higher improvements in I-ANC areas than in S-ANC areas (DID: 14 pp and 7 pp, respectively). The proportions of mothers who received the recommended number of home visits by AWWs (≥3 visits) and ASHAs (≥4 visits), however, were still suboptimal, at 36% and 57%, respectively. Mothers in I-ANC areas were more likely to participate in VHNDs than those in S-ANC areas at endline (59% compared with 48%) and more likely to receive individual (52% compared with 43%) and group counseling (44% compared with 33%).

**TABLE 3:** Coverage and use of ANC services and nutrition interventions among recently delivered women by survey round in I-ANC and S-ANC intervention areas in Uttar Pradesh, India^1^

	Baseline		Endline		
	I-ANC area (*n* = 904)	S-ANC area (*n* = 934)	I-ANC area^2^ (*n* = 922)	S-ANC area (*n* = 927)	DIDs,^3^ pp ± SE
Received ANC services					
Received any ANC, %	91.0	89.7	89.8	88.4	−0.1 ± 2.1
Received ANC from first trimester, %	41.0	38.1	41.1	44.0	−5.9 ± 3.5
Received ANC ≥4 times, %	29.4	24.3	23.5	22.7	−4.8 ± 3.4
Home visits					
Visited at home by AWW, %	27.0	32.0	61.6**	51.4	14.4 ± 4.3**
Received ≥3 visits at home by AWW, %	15.2	15.5	36.2*	27.8	8.4 ± 3.5*
Visited at home by ASHA, %	80.1	83.5	90.6	86.7	6.8 ± 2.6*
Received ≥4 visits at home by ASHA, %	40.7	40.9	56.5*	49.4	6.6 ± 3.7
Received ≥7 visits at home by AWW or ASHA, %	18.7	18.0	38.9*	31.7	6.2 ± 3.4
VHNDs					
Participated in VHNDs, %	—	—	59.2**	48.3	—
Times attended VHNDs in the last 3 mo, *n*	—	—	1.4 ± 1.5**	1.1 ± 1.3	—
Received any individual counseling, %	—	—	52.2*	42.9	—
Participated in any group counseling, %	—	—	44.2**	33.0	—
Received counseling on maternal nutrition and breastfeeding practices					
Eating variety of foods (5 groups), %	51.2	51.7	64.0***	54.6	9.9 ± 4.1*
Increasing quantities of foods, %	32.0	37.3	69.3***	51.0	23.2 ± 3.5***
Weight gain, %	17.9	19.8	50.3**	40.2	11.7 ± 3.7***
Taking IFA, %	70.8	73.7	89.4***	80.9	11.0 ± 3.7***
Taking calcium, %	19.7	20.9	68.0***	54.6	14.0 ± 3.7***
Breastfeeding, %	36.1	43.4	63.1**	53.9	15.5 ± 4.1***

1 Values are means ± SDs or percentages. ANC, antenatal care; ASHA, Accredited Social Health Activist; AWW, Anganwadi worker; DID, difference-in-difference effect estimate; I-ANC, nutrition-intensified antenatal care; IFA, iron and folic acid; pp, percentage points; S-ANC, standard antenatal care; VHND, Village Health and Nutrition Day.

2 Differences in groups at baseline and endline: *P < 0.05, **P < 0.01, ***P < 0.001.

3 DIDs between baseline and endline: *P < 0.05, **P < 0.01, ***P < 0.001.

Overall exposure to counseling from any platform or any service provider improved in both areas over time, with improvements in I-ANC compared with S-ANC areas for counseling on diet diversity (effect: 10 pp), adequate intake (effect: 23 pp), IFA (effect: 11 pp) and calcium supplement consumption (effect: 14 pp), weight gain (effect: 12 pp), and breastfeeding (effect: 16 pp). Detailed contents of counseling for each topic were ∼5–10 pp higher in I-ANC than in S-ANC areas (**Supplemental Table 2**).

### Effects on receipt and consumption of IFA and calcium supplements

**FIGURE 2:**
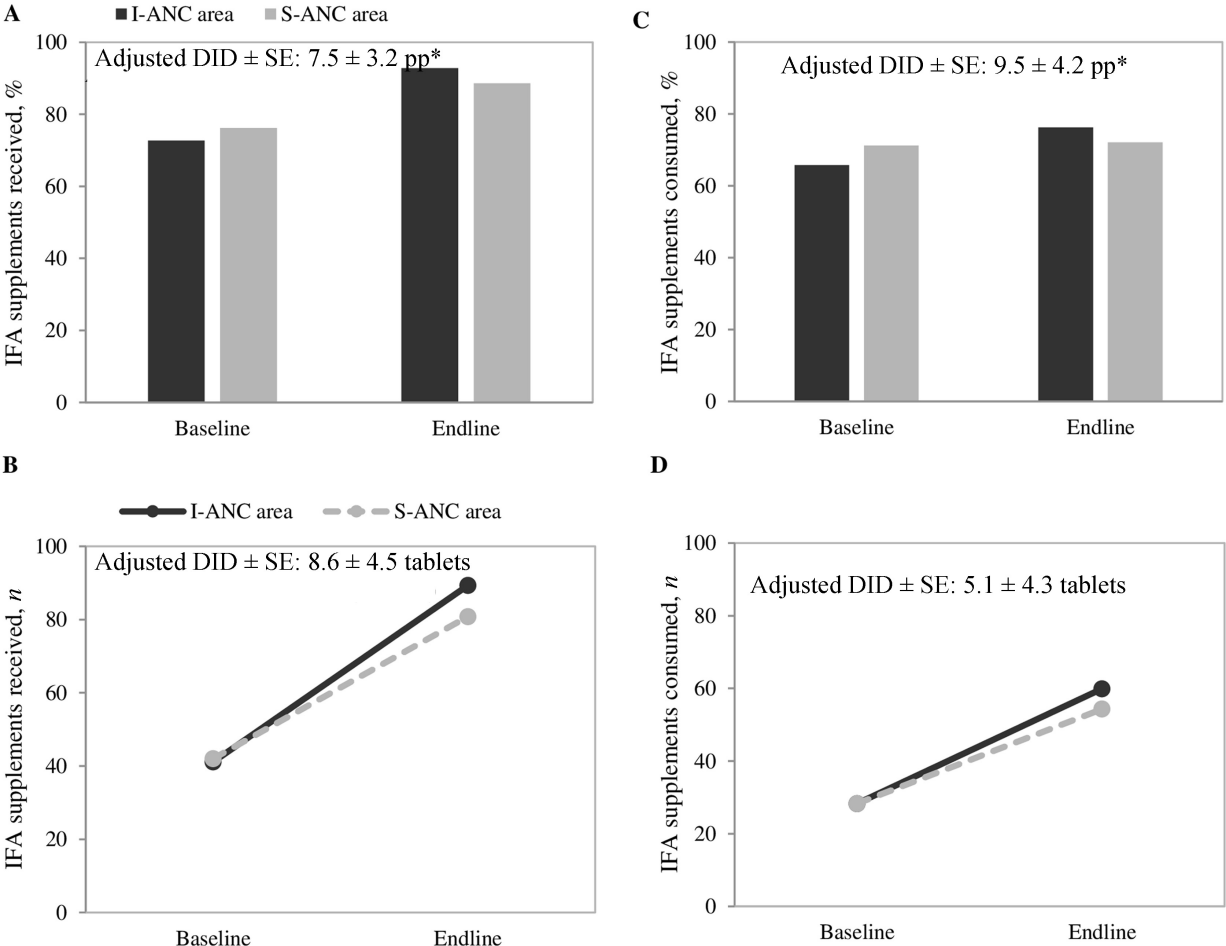
Receipt and consumption of IFA supplements in recently delivered women, by survey round in I-ANC and S-ANC intervention areas in Uttar Pradesh, India. (A) Percentage of women that received IFA supplements, (B) number of IFA supplements received, (C) percentage of women that consumed IFA supplements, (D) number of IFA supplements consumed. *Significant difference: *P* < 0.05. Percentage that consumed ≥100 IFA tablets: 8.0% compared with 7.0% at baseline and 26.0% compared with 23.2% at endline for I-ANC and S-ANC, respectively. DID, difference-in-difference effect estimate; I-ANC, nutrition-intensified antenatal care; IFA, iron–folic acid; pp, percentage points; S-ANC, standard antenatal care.

Receipt of IFA supplements improved in both areas over time, with higher improvements in I-ANC areas than in S-ANC areas (effect: 7.5 pp) (**Figure 2**). The number of IFA supplements received at endline also doubled from baseline, with a mean of 89 tablets in I-ANC areas compared with 81 tablets in S-ANC areas. The proportion of women who consumed IFA supplements increased over time in I-ANC areas but remained unchanged in S-ANC areas, yielding an impact of 9.5 pp. Although the number of IFA tablets consumed improved in both areas over time, overall consumption remained far from optimal during pregnancy (<60 tablets). Consumption of ≥100 IFA tablets was nondifferential and low in both areas (27% in I-ANC and 23% in S-ANC areas).

**FIGURE 3:**
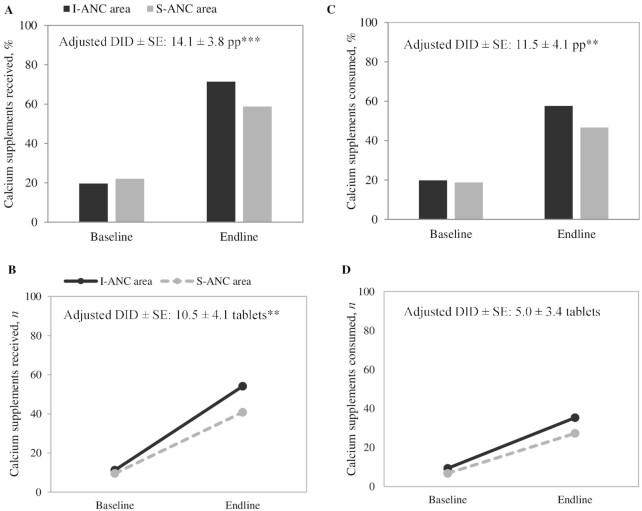
Receipt and consumption of calcium supplements in recently delivered women, by survey round in I-ANC and S-ANC intervention areas in Uttar Pradesh, India. (A) Percentage of women that received calcium supplements, (B) number of calcium supplements received, (C) percentage of women that consumed calcium supplements, (D) number of calcium supplements consumed. ^**,***^Significant difference: **P < 0.01, ***P < 0.001. Percentage that consumed ≥100 calcium tablets: 3.4% compared with 1.8% at baseline and 13.5% compared with 10.4% at endline for I-ANC and S-ANC, respectively. DID, difference-in-difference effect estimate; I-ANC, nutrition-intensified antenatal care; pp, percentage points; S-ANC, standard antenatal care.

For calcium supplements, improvements were greater in the I-ANC arm than in the S-ANC arm for receipt (effect: 14 pp, 11 tablets) and consumption (effect: 11.5 pp) (**Figure 3**). The numbers of calcium tablets received (41–54 tablets) and consumed (27–35 tablets) were low compared with the recommended calcium consumption of 360 tablets during pregnancy.

Repeated-measures longitudinal analyses also found modestly greater improvements in the numbers of IFA tablets received (21) and consumed (16), and the proportion of women consuming ≥100 IFA tablets (10 pp), for I-ANC than for S-ANC areas (**Supplemental Table 3**). Similar findings were shown for calcium supplements received and consumed (6 and 5 tablets, respectively).

### Effects on dietary diversity and micronutrient adequacy

Repeated cross-sectional analyses showed that diet diversity improved similarly in both arms over time, but less than half of pregnant women consumed ≥5 food groups in the previous 24 h (**Table 4**). MPA of micronutrients was low (∼20%) and similar in both arms over time. Probability of adequate intake was also very low for several micronutrients, including key ones such as iron and calcium.

**TABLE 4:** Diet diversity and probability of adequate intake of micronutrients among pregnant women by survey round in I-ANC and S-ANC intervention areas in Uttar Pradesh, India^1^

	Baseline		Endline		
	I-ANC area^2^ (*n* = 333)	S-ANC area (*n* = 334)	I-ANC area (*n* = 340)	S-ANC area (*n* = 334)	DIDs, pp ± SE
Core indicators					
Food groups consumed, *n*	4.2 ± 1.1	4.1 ± 1.1	4.4 ± 1.2	4.3 ± 1.1	0.003 ± 0.1
Consumed ≥5 food groups, %	36.2	34.9	45.2	42.4	1.8 ± 5.5
Mean probability of adequacy of micronutrients, %	17.6	19.3	21.0	21.5	0.8 ± 1.7
Optional indicators					
Type of food groups consumed, %					
All starchy staple foods	100	100	99.7	100	−0.3 ± 0.3
Pulses	59.7	56.1	59.3	58.5	−2.1 ± 5.1
Nuts and seeds	18.1	17.9	18.6	21.8	−4.0 ± 4.7
Dairy	80.6	81.1	82.9	86.2	−3.0 ± 3.7
Flesh foods	3.5	5.1	6.3	5.0	3.5 ± 2.7
Eggs	2.9	1.9	3.9	2.9	−0.01 ± 1.7
Dark green leafy vegetables	38.4	37.2	38.9	32.1	5.5 ± 5.7
Other vitamin A–rich fruits and vegetables	2.5	2.9	3.9	4.7	−0.3 ± 2.0
Other vegetables	77.1	78.8	86.2	86.2	2.1 ± 4.5
Other fruits	32.7	29.2	38.0	35.6	−1.1 ± 5.5
Probability of adequate intake, %					
Calcium	24.4	26.4	31.9	32.0	1.2 ± 2.6
Iron	3.0	3.7	3.1	4.9	−1.4 ± 1.6
Zinc	49.3	52.4	54.5	52.8	4.1 ± 4.3
Vitamin C	11.3	16.9	18.3	21.7	2.1 ± 4.2
Thiamin	53.0	56.0	57.8	57.1	2.7 ± 4.4
Riboflavin	14.4	15.7	16.6	17.6	−0.3 ± 3.5
Niacin	34.9	38.0	39.2	40.6	1.2 ± 4.4
Vitamin B-6	0.9	0.9	2.2	1.7	0.5 ± 1.0
Folate	1.2	0.5	1.9	1.6	−0.4 ± 1.1
Vitamin B-12	1.1	1.4	5.1	5.9	−0.7 ± 1.7
Vitamin A	0.2	0.1	0.1	0.2	−0.1 ± 0.1

1 Values are means ± SDs or percentages. No comparisons were statistically significant. DID, difference-in-difference effect estimate; I-ANC, nutrition-intensified antenatal care; pp, percentage points; S-ANC, standard antenatal care.

The repeated-measures longitudinal analyses showed similar patterns of food group consumption and dietary diversity, but women in I-ANC areas had slightly higher MPA (3 pp), higher consumption of vitamin A–rich foods (10 pp, 11 g/d), higher quantity of other vegetables and other fruits (22–29 g/d), and higher thiamin (13 pp) and niacin (9 pp) than those in S-ANC areas (Supplemental Table 3).

### Effects on weight monitoring and weight gain

**FIGURE 4:**
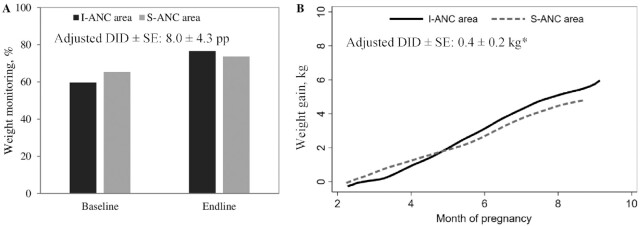
Weight monitoring during pregnancy and gestational weight gain, by survey round in I-ANC and S-ANC intervention areas in Uttar Pradesh, India. (A) Weight monitoring, (B) gestational weight gain. *Significant difference: *P* < 0.05. DID, difference-in-difference effect estimate; I-ANC, nutrition-intensified antenatal care; pp, percentage points; S-ANC, standard antenatal care.

Weight gain monitoring improved similarly in both areas from baseline (**Figure 4**). At endline, ∼75% of women reported ever being weighed, with weight monitored ∼2 times during pregnancy on average. Mean weight gain for all women was ∼5 kg, much lower than the recommended gestational weight gain for underweight or normal-weight women (10–12 kg) (37).

The repeated-measures longitudinal analyses showed that women in I-ANC areas had modestly higher gestational weight gain (0.4 kg) than those in S-ANC areas (Supplemental Table 3).

### Effects on breastfeeding practices

**FIGURE 5:**
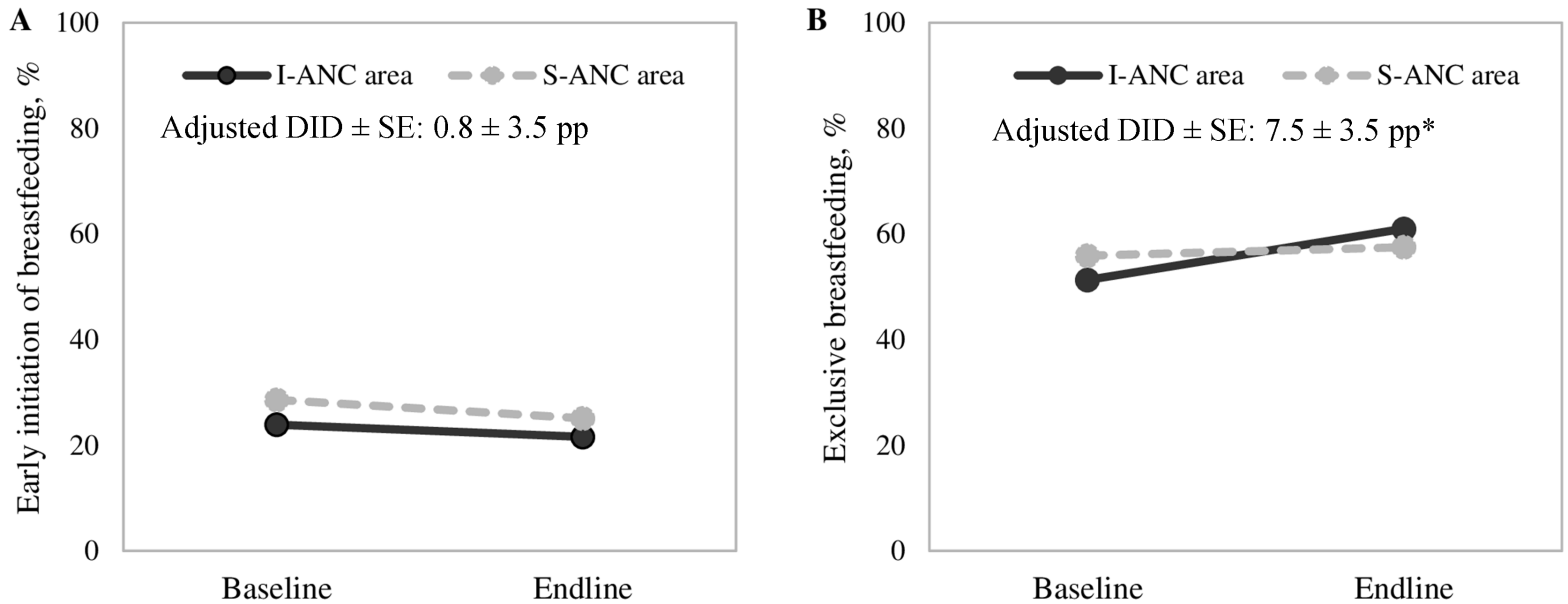
Breastfeeding practices, by survey round in I-ANC and S-ANC intervention areas in Uttar Pradesh, India. (A) Early initiation of breastfeeding, (B) exclusive breastfeeding. *Significant difference: *P* < 0.05. DID, difference-in-difference effect estimate; I-ANC, nutrition-intensified antenatal care; pp, percentage points; S-ANC, standard antenatal care.

I-ANC had a modest effect on the proportion of mothers who reported exclusively breastfeeding their infants 0–6 mo of age (effect: 7.5 pp), which reached 61% at endline (**Figure 5**). Early initiation of breastfeeding remained low in both areas (22%–25%).

## Discussion

The intervention package of strengthening delivery of micronutrient supplements and intensifying interpersonal counseling and community mobilization implemented through government ANC services had modest impacts on receipt and consumption of IFA and calcium supplements, consumption of vitamin A–rich fruits and other vegetables, gestational weight gain, and exclusive breastfeeding. Although dietary diversity improved over time, there was no differential impact. Early initiation of breastfeeding remained low (<25%) and unchanged over time. Several forms of service provision (home visits by FLWs and counseling) and maternal nutrition practices (receipt and consumption of IFA supplements, dietary diversity, and weight gain monitoring) improved in both areas over time.

The direction of the effects of the intervention package on improving the proportion of women who consumed IFA and calcium supplements is consistent with a previous study in Bangladesh (10–13 pp) (38) and with literature on IFA distribution through ANC (39). The magnitude of the impact was substantially lower in our study than in the Bangladesh study when considering the number of tablets consumed (∼5 tablets compared with 50 tablets) (38). In I-ANC areas, most pregnant women (∼90%) received IFA tablets and three-quarters reported consuming some IFA supplements, but few consumed enough. On average, women only consumed 60 tablets and only one-quarter consumed ≥100 IFA tablets during pregnancy. The numbers of calcium tablets consumed were even lower at 35. Although the program was able to increase awareness on use of supplements through nutrition education, challenges related to adherence, supply chain constraints, and family support remained. The challenges in adherence were aligned with the 4 sequential falter points—ANC attendance, IFA receipt or purchase, IFA consumption, and the number of tablets consumed—reported in several other countries (39). Similar challenges were also experienced in studies from Bihar (40) and Indonesia (41). Systems-level factors which may influence intervention efforts include high numbers of vacancies among FLWs and supervisors, a weak supportive supervision system, and inadequate procurement and supply chain management (42). Other supply challenges were identified in our qualitative study including stock-outs, poor demand estimation, lack of storage facilities, and unsystematic distribution (43). Owing to unawareness of the supplements and limited knowledge on their benefits, husbands, mothers, and mothers-in-law did not fully support the use of supplements and did not remind pregnant women to take them (43).

For dietary diversity, we found modest impacts on fruit and vegetable intakes but not on overall dietary diversity. Previous literature has shown impacts of nutrition education counseling on maternal dietary intake in Bangladesh (1.6 food groups) (38) or overall intakes of energy, protein, and micronutrients (44, 45). The reason for the higher impact in Bangladesh could be that more time for counseling was available when providing it through a nongovernmental organization (Bangladesh) than when working through the government system (India). In addition, identified reasons for these limited impacts on dietary diversity in Uttar Pradesh include limited resources (where the nutritious diets that met nutrient requirements were unattainable for most households) and habitual diet-related behavior (including food taboos and diet preferences reducing consumption of animal-source foods) (46). A cost-of-diet analysis included in the overall evaluation showed that home production and social protection strategies could help to reduce the affordability gap for obtaining a nutritious diet (46). Experiences from other countries also bear out the need for additional support to achieve dietary impacts, including a food-assisted maternal and child health and nutrition program that showed positive effects on maternal dietary diversity in Burundi (47) and a homestead food production program that found impacts on consumption of micronutrient-rich foods in Cambodia (48).

Although the intervention showed modest impact on gestational weight gain, mean weight gain for all women was low (∼5 kg), much lower than the recommended gestational weight gain of 10–12 kg. Gestational weight gain is influenced by many factors including prepregnancy BMI, parity, socioeconomic status, ANC care and diet during pregnancy, and comorbid medical conditions (49). Women in our study areas had low prepregnancy BMI and high prevalence of underweight (∼20%), thus they entered pregnancy with an unfavorable nutritional condition and intervening during pregnancy may not be early enough. Earlier interventions from the preconception period have been recommended given the large potential health return and relatively low costs and risk of harm (50, 51). During pregnancy, although I-ANC had positive impacts on home visits and counseling on core nutrition messages, many women in the I-ANC area still did not receive the recommended interventions (only about half of women received early ANC, one-fourth received ≥4 ANC visits, and just over one-third received the recommended number of home visits from FLWs). Suboptimal exposure to key platforms together with resource constraints likely explain the limited impact on weight gain and other maternal nutrition practices such as dietary intake and consumption of micronutrient supplements.

The effect on exclusive breastfeeding was similar to results from systematic reviews of breastfeeding promotion interventions (52, 53), but the magnitude was smaller than for previous studies in Bangladesh (38). Some challenges on the demand side were highlighted including social norms on giving prelacteals immediately after birth or discouraging breastfeeding in certain situations. Like our study, studies from Bangladesh and Vietnam did not find effects for early initiation of breastfeeding, highlighting the importance of support immediately after birth to improve this practice (54).

In addition to modest improvements in I-ANC areas, we observed dramatic secular improvements over time in both areas for both service provision and behavior change. The secular change was likely due to health systems and implementation improvements resulting from the national programs which were implemented concurrently, including the National Nutrition Mission (POSHAN Abhiyaan) and the Anemia-Free India campaign, among others. At the implementation level, A&T's presence led to some spillover to S-ANC areas (19), including sharing of project learnings at district nutrition meetings, endorsement of A&T SBCC materials by government, sharing of counseling practices/tools on FLW district-level WhatsApp groups, and sharing of audio-visual materials from VHNDs and community-based events. A&T materials were accessed by ∼95% of FLWs in I-ANC areas and ∼50% in S-ANC areas (42). On the supply chain, some spillover may also have accrued owing to advocacy and technical support by A&T and partners to improve the procurement and management of IFA and calcium supplements at the national/state level and technical assistance with stock monitoring and forecasting at the district/block level. Overall, these considerations reflect some of the realities of managing a randomized program evaluation in a rapidly evolving policy and program context, but the improvements in the I-ANC area highlight the relevance of focused and local system-strengthening efforts even in the context of broad-scale national, state-, or district-wide program improvements.

The findings should also be interpreted in the context of the overall performance of public health systems in Uttar Pradesh. Previous research has highlighted many of the challenges facing the state health and nutrition programs including systems-related issues, logistical gaps, resource scarcity, and poor utilization of services (42). Indeed, program monitoring data collected during the intervention period highlighted 30%–40% vacancy rates in ANM positions and inadequate stock of IFA and calcium supplements in most blocks (30%–50% of FLWs reported stock-outs in the months before the endline survey) (42). Qualitative interviews conducted with FLWs and supervisors during the process evaluation also highlighted supply chain constraints for IFA and calcium supplements, lack of human resources and equipment to provide services, coordination issues between different FLWs and government departments, and overtasked FLWs (43). Finally, the intervention was limited to ∼18 mo and underwent operational challenges (e.g., supervisor and FLW vacancies, challenges in coordination among FLWs from different departments, and overtasked FLWs) (55) and adaptations within this period to support adequate implementation. Our findings of modest impact on most primary outcomes are a reminder that longer program durations are likely needed to address behavior change effectively, especially in contexts of prevailing system-level challenges.

A recent evidence review on maternal nutrition counseling has highlighted concerns of lack of information related to the coverage and quality of counseling, specifically the format, duration, and frequency of counseling; content of health worker training; and supportive supervision (11). Our study bridged key evidence gaps by including details of timing and frequency of contacts by service providers, duration and content of counseling, and mode of interactions through home visits and community events. The intervention package included capacity-building activities such as trainings of FLWs, performance improvement strategies through supportive supervision, and service delivery support through materials and job aid. Our study used both a repeated cross-sectional design and a repeated-measures longitudinal study to assess the impacts of I-ANC. The repeated cross-sectional design focused on public health and implementation systems impact but precluded the ability to fully link individual exposure to program interventions during pregnancy to outcomes for the same individual as a mother. Using the repeated-measures longitudinal design, we were able to track the exposure and outcomes for the same women from the first trimester until 42 d postpartum. The 2 study designs demonstrated similar results, which confirms the robustness of the findings, suggesting that the repeated cross-sectional design is more feasible and practical for a large-scale programmatic setting. As with other behavioral research, most outcomes were based on maternal recall which is subject to recall bias and social desirability in reporting, but we found no evidence of intervention-specific differentials in socially desirable reporting for maternal nutrition practices.

In conclusion, this study demonstrated the feasibility and value of interventions to strengthen the integration of nutrition interventions into the local-level government ANC services, even in the context of evolving and ongoing national and state-level program improvements during the intervention period. The impacts observed were important but inadequate to achieve desired service delivery and uptake. Promoting and supporting change are possible but strengthening the delivery and use of maternal nutrition services integrated into ANC services in the health system is needed.

## Supplementary Material

nxab131_Supplement_File

## Notes

Supported by the Bill & Melinda Gates Foundation, through Alive & Thrive (A&T), managed by FHI Solutions (to PM). Also supported by the CGIAR Research Program on Agriculture for Nutrition and Health (A4NH), led by the International Food Policy Research Institute (IFPRI).

Author disclosures: the authors report no conflicts of interest.

SG, PKS, JE-A, and TF were members of the program implementation team that designed and implemented the interventions studied and reported on in this article. They reviewed the manuscript and provided contextual interpretation of the results, but final decisions for manuscript content lay with the primary authors from the evaluation team (PHN, SK, LMT, EAF, and PM).

Supplemental Tables 1–3 and Supplemental Figure 1 are available from the “Supplementary data” link in the online posting of the article and from the same link in the online table of contents at https://academic.oup.com/jn/.

Abbreviations used: ANC, antenatal care; ANM, Auxiliary Nurse Midwife; ASHA, Accredited Social Health Activist; AWW, Anganwadi worker; A&T, Alive & Thrive; DID, difference-in-difference effect estimate; FLW, frontline worker; I-ANC, nutrition-intensified antenatal care; ICDS, Integrated Child Development Services; IFA, iron–folic acid; ILA, incremental learning approach; MPA, mean probability of adequacy; NFHS, National Family Health Survey; pp, percentage points; RMNCH+A, Reproductive, Maternal, Neonatal, Child, and Adolescent Health; S-ANC, standard antenatal care; SBCC, social and behavior change communication; VHND, Village Health and Nutrition Day.
