# Supplementary material for: Strengthening Nutrition Interventions in Antenatal Care Services Affects Dietary Intake, Micronutrient Intake, Gestational Weight Gain, and Breastfeeding in Uttar Pradesh, India: Results of a Cluster-Randomized Program Evaluation
Source: J Nutr. 2021 May 26;151(8):2282–95. doi: 10.1093/jn/nxab131 (PMC8349122; doi:10.1093/jn/nxab131)
Supplement: nxab131_Supplement_File [file nxab131_supplement_file.pdf]

Nguyen et al. (2021). Strengthening Nutrition Interventions in Antenatal Care Services Affects Dietary Intake, Micronutrient Intake, Gestational Weight Gain, and Breastfeeding in Uttar Pradesh, India: Results of a Cluster-Randomized Program Evaluation.
On-line Supplemental Material.

Supplemental Table 1: Social desirability bias[1,2] by program arm

	**I-ANC**	**S-ANC**
**Pregnant women**	***n* = 334**	***n* = 340**
Mean, *n*	7.8 ± 2.2	8.0 ± 2.0
Low (0-5), *%*	15.9	10.9
Medium (6-7) , *%*	22.8	22.1
High (8-9) , *%*	40.1	45.9
Very high (10-12) , *%*	21.3	21.2
**RDW**	***n* = 922**	***n* = 927**
Mean, *n*	7.6 ± 2.3	7.6 ± 2.2
Low (0-5) , *%*	19.1	19.6
Medium (6-7) , *%*	21.9	19.3
High (8-9) , *%*	41.9	45.9
Very high (10-12) , *%*	17.1	15.2

[1]Social desirability is the tendency of respondents to answer questions in a manner that is viewed favorably by others. Recognizing the potential role of social desirability in influencing reporting of nutrition behaviors, we examined potential bias from social desirability in our impact analysis. We administered the following 12 questions with yes or no response to assess social desirability bias:

1. Do you sometimes find it hard to go on with your work if you are not encouraged?
2. Do you sometimes feel resentful when you don't get your way?
3. Do you occasionally give up doing something because you don't think you have the ability?
4. Are there any times when you felt like rebelling against people in authority even though you knew they were right?
5. Are you always a good listener no matter who you are talking to?
6. Are there any occasions when you took advantage of someone?
7. When you make a mistake, are you always willing to admit it?
8. Are you always courteous, even to people who are disagreeable/not pleasant?
9. Have you ever been irked when people expressed ideas very different from your own?
10. Are there any times when you were quite jealous of the good fortune of others?
11. Do you sometimes get irritated/annoyed by people who ask you to do something for them?
12. Have you ever deliberately said something that hurt someone's feelings?

The social desirability score was created by adding up the number of socially desirable answers, thus it ranges from 0-12, with a score of 0-5 being considered as a low score, 6-7 as medium score, 8-9 as high, and 10-12 as very high. The key assumption is that respondents who answer in a socially desirable manner on the scale may also respond to other questions (e.g., those related to dietary diversity, micronutrient consumption or IYCF practices) in a manner they think is socially desirable.

[2]Values are means ± SDs or percentages.

Nguyen et al. (2021). Strengthening Nutrition Interventions in Antenatal Care Services Affects Dietary Intake, Micronutrient Intake, Gestational Weight Gain, and Breastfeeding in Uttar Pradesh, India: Results of a Cluster-Randomized Program Evaluation.
On-line Supplemental Material.

Supplemental Table 2: Detailed counseling messages received among recently delivered women by program arm and survey round[1]

	Baseline		Endline		Difference-in-difference effect estimates[3]
	I-ANC area[2] *n*=904	S-ANC area *n*=934	I-ANC area *n*=922	S-ANC area *n*=927	
**Messages on eating a variety of foods, %**					
Eat five different types of food in addition to roti/rice	20.5	21.3	43.4**	34.7	9.4*
Consume thick daal5 everyday	36.9	36.5	52.9**	44.4	8.0*
Consume milk/ milk product daily	37.3	38.1	47.7*	41.6	6.9
Consume dark green leafy vegetable daily	42.8	43.6	50.8***	42.8	8.8*
Consume yellow/ orange fruit and vegetable daily	29	30.1	41.4***	32.6	10.0**
Consume egg daily, if acceptable	4.6	4.5	11.7**	7.3	4.1*
Consume fish/meat daily, if non-vegetarian	3.2	4.6	11.9**	6.9	6.5***
Why different varieties are required	1.9	2	14.0*	9.8	4.2*
**Messages on quantity of foods, %**					
A woman needs more energy and nutrients during pregnancy and lactation.	6.2	8.5	27.2***	18.6	10.5***
To increase the amount of food depending on the month of pregnancy	4.4	6.4	28.7*	23.3	7.0**
Eat 2 complete meals daily during 1st trimester of pregnancy	1.4	2.2	25.7***	16.5	9.7***
Eat 3 complete meals daily during 2nd trimester of pregnancy	2.4	3.1	22.9***	14.5	8.8***
Eat 3 complete meals with 2 nutritious snacks daily during 3rd trimester of pregnancy	1.7	2.7	18.5***	11.1	8.2***
Eat 3 complete meals everyday with 3 nutritious snacks during lactation	2.2	2.9	18.8***	9.5	9.8***
Why increased quantities of food are needed	7	9.9	13.4	10.8	5.5*
**Messages on IFA supplements, %**					
Take 1 tablet daily during pregnancy	41.9	41.2	20.8	19	1.1
Take 180 IFA tablets during pregnancy	2	2.7	14.5	12.2	2.7
Continue to take 1 tablet/day till 6 mo postpartum	2.9	4.6	15.1	15	1.6
Take IFA with water or lemon water	20.1	20.1	39.2***	28.3	10.7**
Do not take IFA with tea or milk	2.5	1.4	6.2	4.7	0.2
Take IFA at night before bedtime	21.9	24.6	46.6	40.8	8.5*
Do not take IFA and calcium together	1	2	10.2	7.9	3.3
Do not take IFA tablet on an empty stomach	6.5	5.7	18.3	15.2	2.3
IFA prevents anemia	15	16.1	28.1	22.9	6.0
IFA reduces risk of low birth weight baby	2.4	3.3	12.3	9.4	3.6
IFA reduces risk of maternal death due to hemorrhage	1.3	1.9	6.6	5.7	1.6
IFA ensures the best development of the child	1.5	1.5	5.5	4.9	0.5
IFA reduces complication pregnancy and birth	2.9	3.2	5.3	4.9	0.7

**Messages on calcium supplements, %**					
Take 1 – 2 tablets daily during pregnancy	9.3	9.5	20.1*	14.9	5.1*
Take 360 calcium tablets during pregnancy	0.4	0.9	10	7.2	2.9
Continue to take 1-2 tablets/day till 6 months postpartum	1.7	1.8	9.3	8.3	1.1
Do not take IFA and calcium together	2.7	1.9	23.2*	17.0	4.9*
Do not take calcium tablet on an empty stomach	2.5	3.3	20.1	17.2	3.4
Take the 1st calcium tablet after breakfast and the 2nd tablet with lunch	2.5	2.4	19.6	18	1.4
Calcium helps in the development of bone and teeth of the baby	3.8	2.2	25.1***	16.8	6.4*
Calcium reduce risk of high blood pressure	1.7	1.9	11.6***	6.1	5.6**
**Messages on weight monitoring and weight gain during pregnancy, %**					
Women should weigh themselves regularly during pregnancy	3.2	3	17.8*	13.4	4.1
Record weight in MCP card	1.2	1.6	11.5	10	1.8
Women should gain 1.5-2 kg/month from the 4th month of pregnancy	1.5	2.2	20.6*	15.1	6.1
Women should gain 9-11kg weight during pregnancy	1.2	2	13.2	9.9	4.1
Gaining weight indicates proper growth of the fetus	5	5.9	14.9	12	3.5
Gaining weight indicates mother is taking adequate food	6.5	7.2	15.2	12.6	2.8
**Messages on breastfeeding, %**					
Initiate breastfeeding within the first hour of birth	20	23	29.2	24.6	7.2
Feed colostrum	5.2	7.9	12.4	10	4.8
Do not put anything in child’s mouth after birth	3.5	3.9	10.5	7.7	3.1
Feed only breastmilk to child for 6 months after birth	9.8	15.1	14.0	13.2	5.8*
Feed expressed breast milk	0.1	0.5	1.3	1.7	-0.04

[1]Values are means ± SDs or percentages; [2]Differences in groups at baseline and endline; [3]Difference-in-difference effect estimates between baseline and endline; *P< 0.05, ** P < 0.01, *** P < 0.001; ANC: Antenatal care; ASHA: Accredited Social Health Activist; AWW; Anganwadi Worker; I-ANC: Intensive antenatal care; IFA: Iron and folic acid; S-ANC: Standard antenatal care; VHND: Village health and nutrition day; [5]daal is a thick soup-like dish made generally of lentils, beans or peas.

Nguyen et al. (2021). Strengthening Nutrition Interventions in Antenatal Care Services Affects Dietary Intake, Micronutrient Intake, Gestational Weight Gain, and Breastfeeding in Uttar Pradesh, India: Results of a Cluster-Randomized Program Evaluation.
On-line Supplemental Material.

Supplemental Table 3. Results from repeated-measures longitudinal study[1]

	Early pregnancy		Late pregnancy		Difference-in-difference effect estimates[3]
	I-ANC area[2] (*n* = 236)	S-ANC area (*n* = 239)	I-ANC area[2] (*n* = 178)	S-ANC area (*n* = 195)	
**Core indicators**					
Ever received IFA, *%*	41.1	37.7	98.5*	94.6	0.7
Number of IFA received, *n*	18.1 ± 31.1	17.4 ± 31.8	105.1 ± 71.3**	85.2 ± 57.8	20.6***
Ever consumed IFA, *%*	32.2	28.9	97.0**	89.4	5.0
Number of IFA consumed, *n*	4.1 ± 9.3*	2.6 ± 6.9	70.9 ± 60.3***	54.5 ± 52.7	16.0**
Consumed ≥100 IFA tablets, *%*	0.0	0.0	30.9*	21.2	9.9**
Ever received Calcium, *%*	16.5*	10.5	59.7	55.1	-1.8
Number of Calcium received, *n*	3.8 ± 11.8**	1.5 ± 5.2	19.4 ± 28.5**	11.8 ± 19.6	5.5**
Ever consumed Calcium, *%*	13.6*	5.0	54.7	49.5	-3.5
Number of Calcium consumed, *n*	1.5 ± 5.4**	0.3 ± 2.2	15.1 ± 23.6**	9.6 ± 18.4	4.5**
Gestational weight gain, *n*	0.9 ± 2.3	0.7 ± 1.8	5.3 ± 3.0*	4.8 ± 3.0	0.4*
Number of food groups consumed, *n*	4.0 ± 1.1	4.0 ± 1.1	4.0 ± 1.0	3.9 ± 1.0	0.02
Consumed ≥5 food groups, *%*	29.2	29.3	28.8	26.8	2.0
**Average food intake (g/person/day)**					
Cereals and starchy staples, *g*	400.3 ± 207.8	407.8 ± 243.7	423 ± 195.3	390.6 ± 182.6	35.1
Pulses, *g*	30.6 ± 41.7	28.4 ± 41.9	26.9 ± 37.5	29.9 ± 36.4	-5.4
Nuts and seeds, *g*	1.5 ± 6.8	1.9 ± 7.3	1.1 ± 5.6	2.9 ± 11.9	-1.3
Dairy, *g*	130.2 ± 170.1	146.1 ± 223.5	183.2 ± 206.5	177.9 ± 209.4	19.3
Meat, poultry and fish, *g*	3.2 ± 13.3	2.5 ± 11.2	3.1 ± 13.3	2.9 ± 13.3	-0.5
Eggs, *g*	0.7 ± 4.6	0.2 ± 2.4	0.2 ± 2.3	0.2 ± 1.8	-0.5
Dark green leafy vegetables, *g*	6.6 ± 23.5	5.4 ± 19.7	2.1 ± 13.3	4.6 ± 21.9	-3.8
Other vitamin A-rich fruits & vegetables, *g*	8.5 ± 29.8	7.5 ± 25.3	18.0 ± 50.3**	6.1 ± 28.8	10.9*
Other vegetables, *g*	62.0 ± 70.1**	81.3 ± 81.8	76.7 ± 78.8	66.1 ± 60.9	29.2**
Other fruits, *g*	31.5 ± 67.6**	51.5 ± 96.5	37.8 ± 75.1	35.2 ± 65.7	22.4*
**Probability of adequate intake**					
Calcium, *%*	12.9	14.2	21	19.7	2.3
Iron, *%*	0.4*	1.2	0.6	0.6	0.8
Zinc, *%*	37.6	39.3	53.0	48.8	5.4
Vitamin C, *%*	10.7	7.6	3.5	2.2	-1.8
Vitamin B1, *%*	37.6	43.3	60.0	52.0	13.0*
Vitamin B2, *%*	5.1	7.7	7.5	6.0	4.0
Niacin –Vitamin B3, *%*	28.1	32	36.5	30.8	9.1*
Vitamin B6, *%*	0.3**	2.3	0.3	0.5	1.8
Folate total, *%*	1.4	0.9	0.6	0.2	-0.2
Vitamin B12, *%*	0.9	1.2	2.1	2.1	0.3
Vitamin A (RAE)	0	0	0	0	0

Nguyen et al. (2021). Strengthening Nutrition Interventions in Antenatal Care Services Affects Dietary Intake, Micronutrient Intake, Gestational Weight Gain, and Breastfeeding in Uttar Pradesh, India: Results of a Cluster-Randomized Program Evaluation.
On-line Supplemental Material.

[1]Values are means ± SDs or percentages; [2]Differences in groups at baseline and endline; [3]Difference-in-difference effect estimates between baseline and endline; I-ANC: Intensive antenatal care; S-ANC: Standard antenatal care.

Supplemental Figure 1: CONSORT diagram for repeated-measures longitudinal study

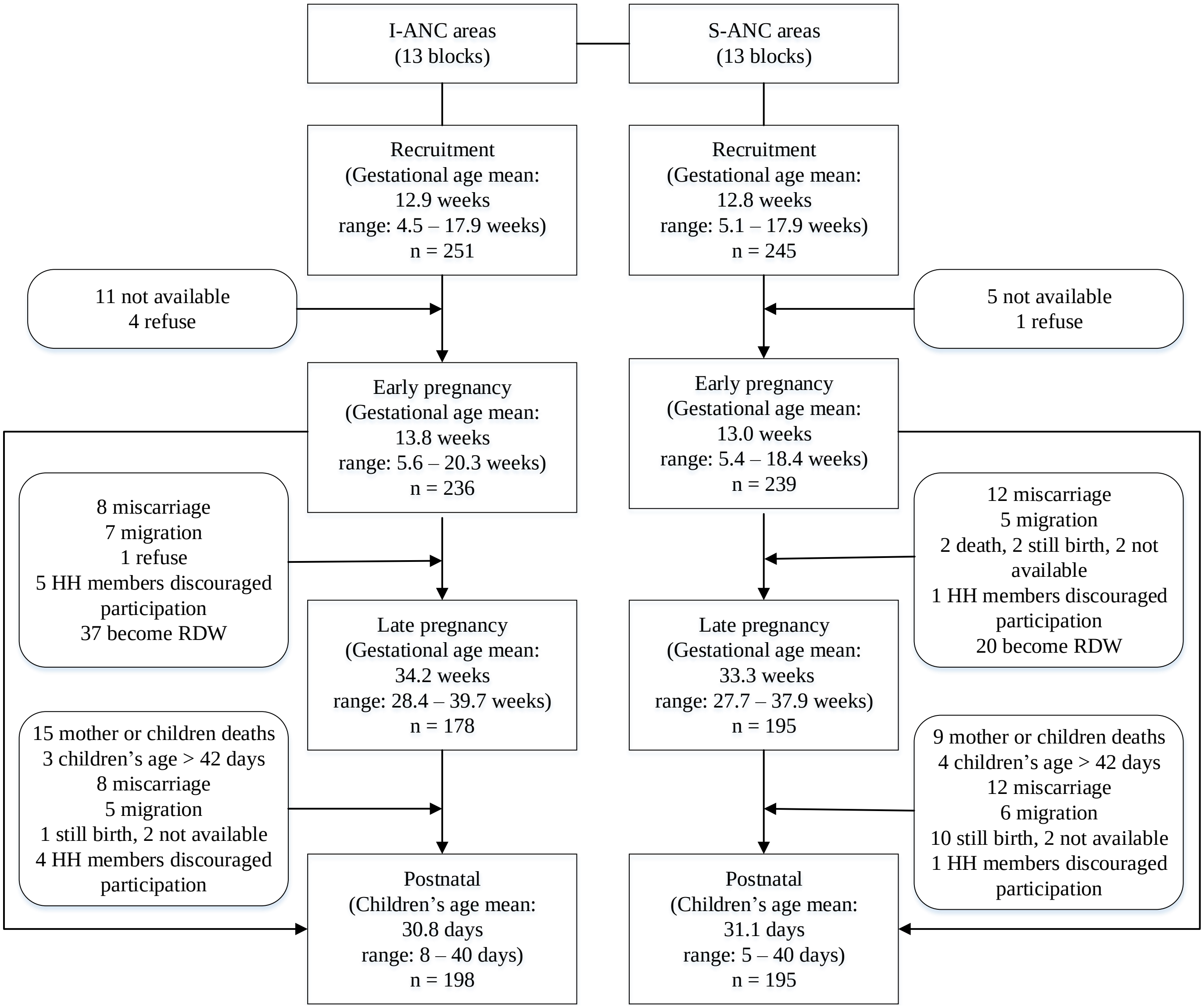

I-ANC: Intensive antenatal care; S-ANC: Standard antenatal care
